# 0033. Hypothermia protects brain mitochondrial function from hypoxia in sepsis

**DOI:** 10.1186/2197-425X-2-S1-O6

**Published:** 2014-09-26

**Authors:** KI Chisholm, AL Davies, M Singer, A Dyson, KK Ida, I Tachtsidis, MR Duchen, KJ Smith

**Affiliations:** University College London, Institute of Neurology, London, UK; University College London, Bloomsbury Institute of Intensive Care Medicine, London, UK; University of São Paulo, Medical School, Anaesthesiology LIM-8, São Paulo, Brazil; University College London, Medical Physics and Bioengineering, London, UK; University College London, Cell and Developmental Biology, London, UK

## Introduction

Hypothermia reduces metabolic requirements and thereby the oxygen supply-demand imbalance arising from hypoxia. Hypothermia has been an effective treatment in several critical care conditions, but the effects of hypothermia on brain mitochondrial dysfunction in sepsis are poorly understood.

## Objectives

To examine the relationship between spontaneous hypothermia in a murine model of sepsis, and changes in inspired oxygen, systemic oxygenation and mitochondrial function in the cerebral cortex.

## Methods

Mice (C57/bl6) were injected intraperitoneally with lipopolysaccharide (LPS) (5 mg/kg; n = 15) or saline (0.01 ml/g; n = 6). Six hours later, the cerebral cortex was exposed and mitochondrial function assessed using the inherent fluorescence of flavin adenine dinucleotide (FAD). The mice spontaneously breathed a sequence of 21% oxygen (room air), 100%, 21%, 15%, 21% and 10% for 5 minutes each. Seven of the endotoxic mice were maintained at their original body temperature post-LPS injection, while eight were maintained at 37°C using a homeothermic heating mat. Systemic oxygenation was assessed using pulse oximetry. Statistical analysis was performed by selecting areas around the veins and arteries in order to ratio the fluorescence intensity of arteries over veins. The difference in the ratio between groups was compared using a one way ANOVA followed by independent sample t-tests on IBM SPSS 22.

## Results

**Figure 1:**
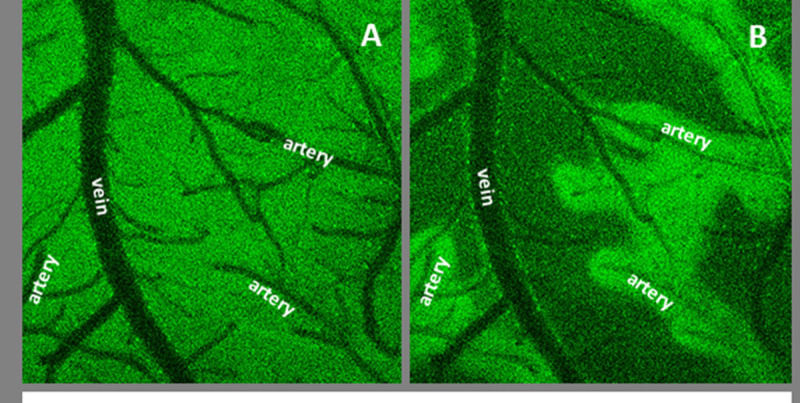
**Example of an arterial halo.** A) Corex of a control mouse subjected to room air and imaged fro FAD. B) Same mouse subjected to 10% inspired oxygen

Hyperoxia (100% O_2_) had little influence on FAD fluorescence. In contrast, hypoxia (≤15% O_2_) resulted in loss of FAD signal (i.e. increased reduced state of the FAD/FADH pool) except for a prominent preservation of signal in a ´halo´ around arteries/arterioles as seen in Figure 1. The threshold for the selective loss of FAD fluorescence appeared ≤10% O_2_ in control mice (maintained at 37.0°C). Loss occurred at higher inspired oxygen (15% O_2_) in normothermic endotoxic mice, than in mice at their spontaneous hypothermic temperature (32.4°C ± 2.0). Arterial oxygen saturation was similar in normothermic control and endotoxic mice breathing 15% O_2_ (endotoxic mice at 37°C = 56.1% ± 6.8, controls at 37°C = 58.1% ± 3.4), but was non-significantly higher in hypothermic endotoxic mice (69.2% ± 12.5).

## Conclusions

Systemic administration of LPS increases the vulnerability of cortical mitochondrial function to reductions in inspired oxygen, despite maintenance of arterial oxygen saturation. This vulnerability was reversed by spontaneous hypothermia.

